# Selected Hematological Biomarkers to Predict Acute Mortality in Emergency Department Patients. Recent Polish Hospital Statistics

**DOI:** 10.1155/2020/8874361

**Published:** 2020-07-16

**Authors:** Katarzyna Brzeźniakiewicz-Janus, Marcus Daniel Lancé, Andrzej Tukiendorf, Tomasz Janus, Mirosław Franków, Joanna Rupa-Matysek, Zuzanna Walkowiak, Lidia Gil

**Affiliations:** ^1^Department of Hematology, Multi-Specialist Hospital Gorzów Wielkopolski, Faculty of Medicine and Health Science, University of Zielona Góra, Gorzów Wielkopolski, Poland; ^2^Department of Anesthesiology, Intensive Care Unit and Perioperative Medicine, Hamad Medical Corporation, Doha, Qatar; ^3^Department of Public Health, Wrocław Medical University, Wrocław, Poland; ^4^Department of Forensic and Clinical Toxicology, Pomeranian Medical University in Szczecin, Szczecin, Poland; ^5^Department of Hematology and Bone Marrow Transplantation, Poznań University of Medical Sciences, Poznań, Poland

## Abstract

**Background:**

Complete blood count (CBC), red cell distribution width (RDW), mean platelet volume (MPV), mean corpuscular volume (MCV), mean cell hemoglobin (MCH), mean cell hemoglobin concentration (MCHC), or platelet (PLT) count are referred as predictors of adverse clinical outcomes in patients. The aim of the research was to identify potential factors of acute mortality in Polish emergency department (ED) patients by using selected hematological biomarkers and routine statistical tools.

**Methods:**

The study presents statistical results on patients who were recently discharged from inpatient facilities within one month prior to the index ED visit. In total, the analysis comprised 14,881 patients with the first RDW, MPV, MCV, MCH, MCHC, or PLT biomarkers' measurements recorded in the emergency department within the years 2016–2019 with a subsequent one month of all-cause mortality observation. The patients were classified with the codes of the International Statistical Classification of Diseases and Related Health Problems after 10^th^ Revision (ICD10).

**Results:**

Based on the analysis of RDW, MPV, MCV, MCH, MCHC, and PLT on acute deaths in patients, we establish strong linear and quadratic relationships between the risk factors under study and the clinical response (*P* < 0.05), however, with different mortality courses and threats. In our statistical analysis, (1) gradient linear relationships were found for RDW and MPV along an entire range of the analyzed biomarkers' measurements, (2) following the quadratic modeling, an increasing risk of death above 95 fL was determined for MCV, and (3) no relation to excess death in ED patients was calculated for MCH, MCHC, and PLT.

**Conclusion:**

The study shows that there are likely relationships between blood counts and expected patient mortality at some time interval from measurements. Up to 1 month of observation since the first measurement of an hematological biomarker, RDW and MPV stand for a strong relationship with acute mortality of patients, whereas MCV, MCH, MCHC, and PLT give the U-shaped association, RDW and MPV can be established as the stronger predictors of early deaths of patients, MCV only in the highest levels (>95 fL), whereas MCH, MCHC, and PLT have no impact on the excess acute mortality in ED patients.

## 1. Background

Hematological biomarkers such as red cell distribution width (RDW), mean platelet volume (MPV), mean corpuscular volume (MCV), mean cell hemoglobin (MCH), mean cell hemoglobin concentration (MCHC), or platelet (PLT) count are machine-calculated parameters derived from a complete blood count (CBC). These values have been demonstrated to be predictors of adverse clinical outcomes in patients.

For example, RDW following the adjusted hazard ratios (HRs) for all-cause mortality was strongly associated with an increased risk of death in middle-aged and older (11,827) adults, i.e., for every 1% increment in RDW, all-cause mortality risk increased by 22% (HR = 1.22, 95% CI: 1.15–1.30, *P* < 0.001) [1]. In addition, RDW was strongly associated with deaths in older adults from cardiovascular disease (CVD) (HR = 1.15, 95% CI: 1.12–1.25), cancer (HR = 1.13, 95% CI: 1.07–1.20), and other causes (adjusted HR = 1.13, 95% CI: 1.07–1.18) [2]. Association between RDW and all-cause mortality in chronic kidney disease (CKD) patients was also reported in [3].

Higher MPV as an indicator of larger and more reactive platelets has been associated with a higher risk of death in hemodialysis (HD) patients (all-cause mortality). Following [4] in nearly 150,000 incident HD patients, higher MPV levels (>11.5 fL) were associated with incrementally elevated death risk. In those patients, it was also shown that an increase in mean platelet volume/platelet count (P) ratio was associated with vascular access failure (VAF) [5]. The authors reported that in multivariate Cox regression analysis, MPV/platelet count ratio remained a significant independent risk factor for VAF, even after adjusting for age, sex, diabetes, coronary artery disease, cerebrovascular disease, and vascular access type (HR = 1.16, 95% CI: 1.11–1.22, *P* < 0.001). MPV/P ratio was demonstrated to be a predictor of adverse outcome in non-ST-segment elevation myocardial infarction (NSTEMI) patients (the ratio is a useful marker to predict a long-term prognosis in NSTEMI patients undergoing percutaneous coronary interventions). Moreover, MPV/P ratio was similar to the Global Registry of Acute Coronary Events (GRACE) score [6] but better than MPV for predicting all-cause mortality. Furthermore, it was easier to calculate than the GRACE score.

Recently, MCV as a measure of the average size of erythrocytes has been associated with mortality in many clinical settings. Elevated MCV (generally >100 fL) is often characteristic of underlying conditions such as nutritional deficiencies, drug, and alcohol use [7], vitamin B12 deficiency [8], certain medications, or bone marrow disorders [9]. In patients with ≥3 chronic kidney disease (CKD) stages, MCV was associated with all-cause mortality, cardiovascular disease mortality, and infection-associated mortality [10]. Other authors [11] applied Cox regression analysis of MCV to predict composite cardiovascular (CV) events in CKD patient as a major confounding factor. Based on the >100 K incident HD patients and those with higher (>98 fL) MCV levels, a higher all-cause cardiovascular and infectious mortality risk was also currently confirmed by [12]. A gradient relationship between increased MCV and deaths associated with cerebral ischemic stroke (CIS) and ischemic heart disease (IHD) was reported in Taiwanese investigators using a large-scale (66,294) population-based study [13]. In addition, [14, 15] reported a positive relation of MCV with clinical response in patients with advanced solid malignancies and gastric cancer after chemotherapy with capecitabine.

We have found the fewest mortality reports for MCH and MCHC. Inverse associations between MCH and MCHC and all-cause mortality were observed among men, but not among women [16]. The study, however, was based on a relatively small number of participants (*n* = 403), and these findings warrant confirmation in further prospective studies conducted on a bigger population. In addition, lower MCHC is associated with poorer outcomes in intensive care unit admitted patients with acute myocardial infarction [17].

PLT count is a prognostic indicator in the general population and the elderly. However, the U-shaped relationship exists between platelet count and mortality (low PLT count is associated with cancer in the general population and high PLT count is associated with CVD) [18]. U-shaped mortality curve associated with platelet count was observed in the New York outpatient population of 36,262 individuals (≥65) [19] and among 131,308 Taiwanese older people (≥65) [20]. This finding was also confirmed by [21] in 3 population-based studies on 40,987 inhabitants of 7 Italian areas and [22] in 1797 international stable chronic obstructive pulmonary disease (COPD) patients within 3-year all-cause mortality observation.

In the above-cited reports, relatively longer follow-ups of subjects were taken to estimate the risk and evaluate mortality. In contrast, acute deaths of such individuals have not been fully addressed.

### 1.1. Aim

In this paper, we present statistical results of an analysis of patients who were recently discharged from inpatient facilities within one month prior to the index emergency department (ED) visit. Using selected hematological biomarkers and routine statistical tools, we predict acute mortality in Polish ED patients.

## 2. Materials

We conducted a single-center retrospective study from the medical records and electronic data in the emergency department (ED) at the 1000-bed public Multi-specialist Hospital in Gorzów Wlkp., Poland. Monthly, the hospital discharges approximately 5000 patients and has 1200 admissions in ED ascertained from the codes of the International Statistical Classification of Diseases and Related Health Problems after 10^th^ Revision (ICD10).

### 2.1. Exposure Ascertainment

The following hematological biomarkers were of interest for our aim: RDW, MPV, MCV, MCH, MCHC, and PLT. Patients were included in the study if they: (1) received care between January 1, 2016, and December 31, 2019, (2) were 18 years old or older, and (3) had at least one biomarker measurement. We examined the data from a total of 23,992 patients with 34,929 laboratory tests. Next, according to ICD10 diagnoses classified in the chapters 1-22 under the abbreviation from A to U, the following ED patients were excluded: O (pregnancy, delivery, and puerperium), P (pertain conditions originating in the perinatal period)—as they did not match the age criterion of being 18 or above, Q (congenital malformations, deformations, and chromosomal aberrations)—due to a very small number of cases, R (symptoms, signs and abnormal clinical test results not classified elsewhere (i.e., patients without diagnosis irrespective of deeper investigations)—on account of inaccuracy of diagnoses, S&T (injuries, poisoning, and other specific effects of external factors)—because of the urgent characteristic of the clinical events, focusing on the remaining ICD10 classes (from A to N), representing *a priori* a “more chronic” nature of the diseases. Then, due to repeated visits of some patients in ED and a subsequent treatment that might affect hematological indices, we examined only the first laboratory tests of biomarkers in the studied period.

Measurements of the blood biomarkers were performed in the hospital Laboratory Unit using Sysmex XN-2000 (Sysmex Corporation, Japan) analytical systems using EDTA-KE/2.7 ml samples.

### 2.2. Outcome Ascertainment

The outcome of interest was all-cause mortality. Patients were considered at risk for mortality from the day after the first hematological biomarker measurement to the first occurrence of death or censoring up to a month prior to the ED visit (the end of study follow-up).

## 3. Methods

Associations of RDW, MPV, MCV, MCH, MCHC, and PLT biomarkers with all-cause acute mortality outcomes were estimated using Poisson regression instead of the “classical” Cox model. To model death rates, analyses of observed versus expected events, often called “person-years” summaries, were calculated, which are well known in epidemiological studies (the Cox model is equivalent to Poisson regression and the similarities of the Cox and rate regression Poisson models are already demonstrated). If the exponentiated coefficient from the Cox model is a hazard ratio (HR), then, this is known from the Poisson model as a standardized mortality ratio (SMR) [23]. In the statistical analysis, SMRs were derived in reference to the base-line lowest biomarkers' category (SMR = 1.00) along with the corresponding 95% confidence intervals. A *P* value of <0.05 was considered statistically significant.

Additionally, we explored potential and the best fitted linear and nonlinear relationships between RDW, MPV, MCV, MCH, MCHC, and PLT and the estimated Poisson SMRs, using linear and quadratic approximations.

Statistical analyses were performed using R statistical platform [24].

## 4. Results

In total, the analysis comprised 14,881 patients with the first RDW, MPV, MCV, MCH, MCHC, and PLT biomarkers' measurements recorded in the emergency department within the years 2016–2019 with a subsequent one month of all-cause mortality observation. The categories of the studied hematological biomarkers with acute death fractions are presented in panel A of Table 1.

The estimated standardized mortality ratios along with the corresponding 95% CIs and *P* values for RDW, MPV, MCV, MCH, MCHC, and PLT hematological biomarkers are reported in panel B of Table 1.

The estimates of linear and quadratic regression coefficients providing the best fitting of the model to data for hematological biomarkers with the corresponding 95% confidence intervals and *P* values following the coefficient of determination (*R*2 statistic) are reported in Table 2.

**Figure 1:**
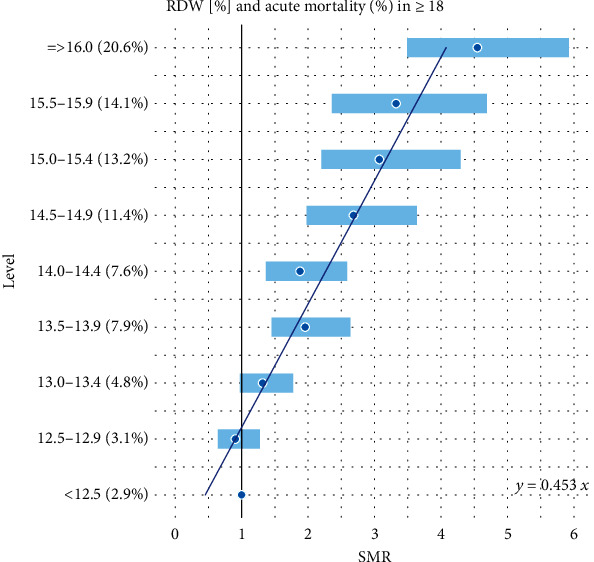
Linear model of SMR of acute (<1 month) mortality in ED patients (≥18) vs. RDW levels.

**Figure 2:**
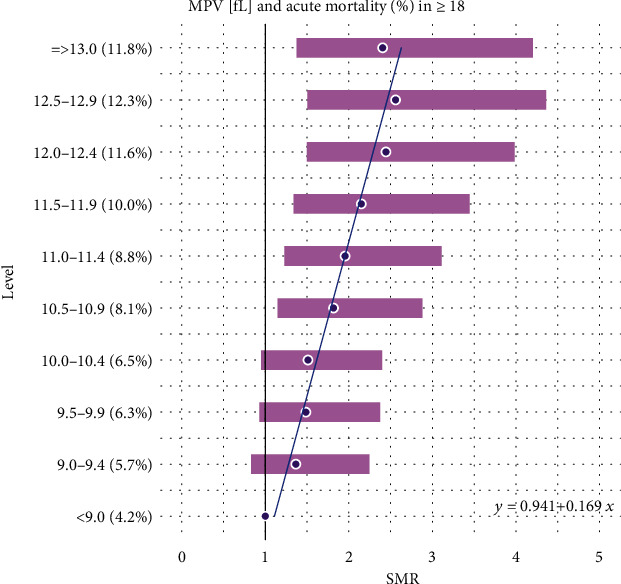
Linear model of SMR of acute (<1 month) mortality in ED patients (≥18) vs. MPV levels.

**Figure 3:**
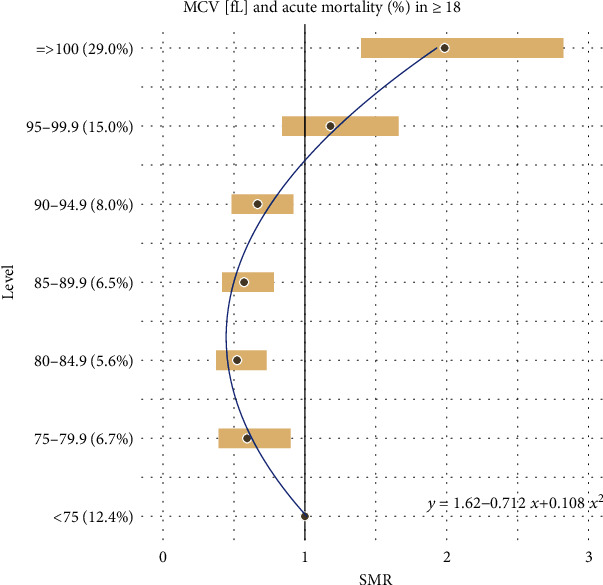
Quadratic model of SMR of acute (<1 month) mortality in ED patients (≥18) vs. MCV levels.

**Figure 4:**
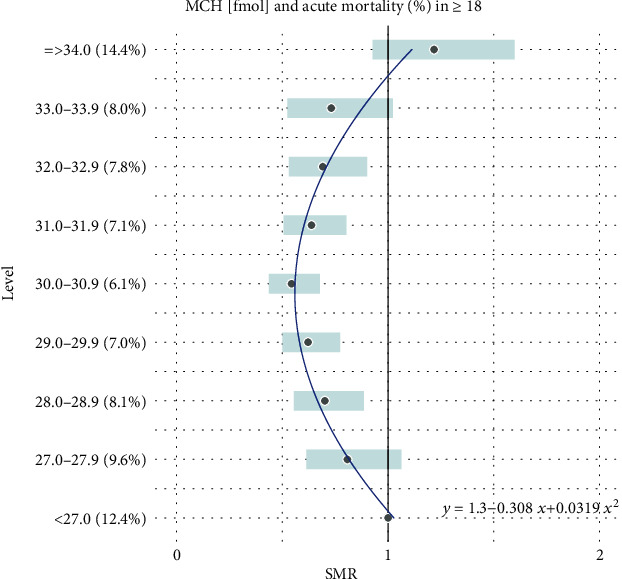
Quadratic model of SMR of acute (<1 month) mortality in ED patients (≥18) vs. MCH levels.

**Figure 5:**
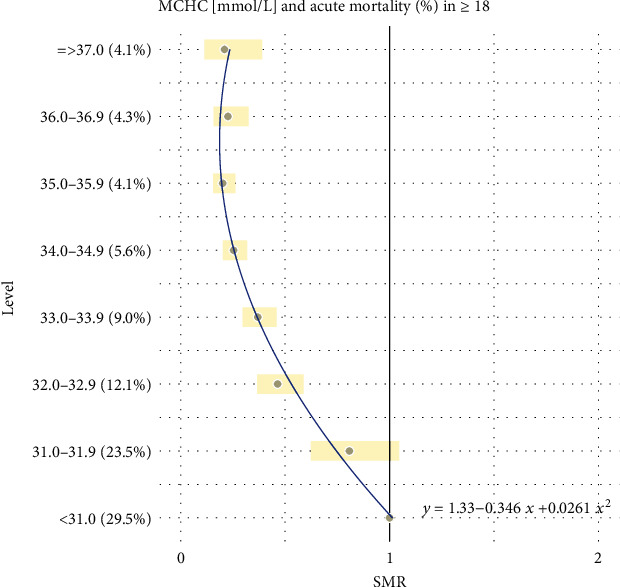
Quadratic model of SMR of acute (<1 month) mortality in ED patients (≥18) vs. MCHC levels.

**Figure 6:**
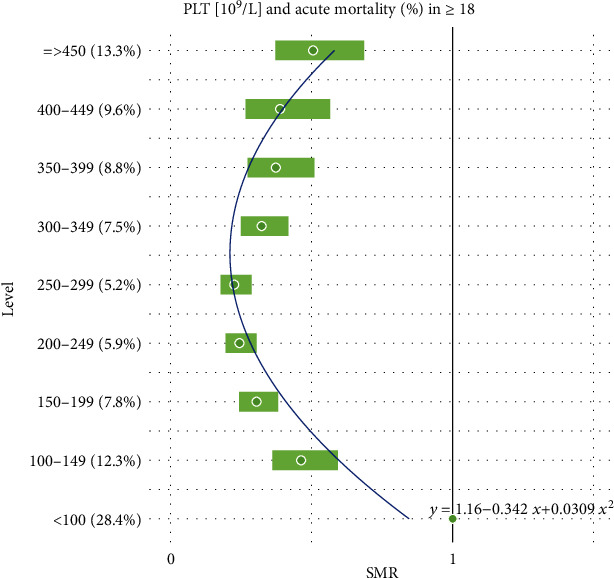
Quadratic model of SMR of acute (<1 month) mortality in ED patients (≥18) vs. PLT levels.

Additionally, all the results reported in Tables 1 and 2 are combined together and presented graphically in Figures 1–6.

## 5. Discussion

The current study shows clearly a positive relationship between the two laboratory values RDW and MPV and the standard mortality rate during the first 31 days. It seems these two results in particular might be predictive of all-cause acute mortality. A relatively large number of samples (nearly 15,000 laboratory tests) may warrant reliable statistical results, and those presented in the paper seem to stand for this belief.

Based on the analysis of RDW, MPV, MCV, MCH, MCHC, and PLT on acute deaths in patients, we established strong linear and quadratic relationships between the risk factors under study and the clinical response (*P* < 0.05), however, with different mortality courses and threats. In our statistical analysis, (1) gradient linear relationships were found for RDW and MPV along with a whole range of the analyzed biomarkers' measurements, (2) following the quadratic modeling, an increasing risk of death above 95 fL was determined for MCV, and (3) no relation to excess death in ED patients was calculated for MCH, MCHC, and PLT.

Many earlier studies confirmed this thesis in other types of patient mortality (the citations will be completed). Association with RDW (Figure 1) obtained in our statistical analysis (*R*^2^ statistic = 0.9866) is almost identical as found by [2] (see Figure 1 of this paper). In the cited work, the authors explain what cause-effect mechanisms between the exposure and the outcome could take place. Furthermore, comparable reports are found about the RDW and mortality. [25] described, in their investigation on 9644 patients coming to a cardiac intensive care unit for admission, an independent association between RDW and hospital mortality. In addition, the RDW seems to be a prognostic marker for 30-day mortality in severe sepsis [26]. So far, for the first time, we have modeled relationships with other hematological biomarkers.

Also, a linear dependence of MPV with acute mortality (Figure 2) looks convincing from a clinical point of view. The reason for this could lie in the nature of some acute diseases which are associated with an increased platelet size. As these are frequently cardiovascular states (i.e., coronary artery disease or stroke), it does not surprise that there are relations between MPV and acute one-month mortality. In a recent meta-analysis by [27], the author describes the MPV being significantly higher in patients with stroke. Similarly, two other groups describe the MPV correlating with the prevalence and the prognosis of coronary artery disease [28, 29]. On the side note, the linearity in our analysis seems convincing and makes these parameters interesting as predictors.

For the remaining hematological biomarkers MCV, MCH, MCHC, and PLT, a much better statistical fitting was made to a set of data on the acute death of patients using a quadratic relationship. However, when it comes to the excess risk of mortality, it was observed only in the case of MCV and only in patients with the highest level of concentration above 95 fL. Moreover, for MCH, MCHC, and PLT, the square curves do not exceed the reference SMR = 1. Therefore, the negative impact of these hematological biomarkers on the risk of acute deaths of patients cannot be confirmed. However, one could use them to exclude a severe course of disease.

Summarizing this section, we realize that a linear regression must point to evident relationship in comparison with a quadratic model due to the shapes of the fitted functions. The question arises why RDW and MPV stand for a strong cause-and-effect association with acute mortality of patients and MCV, MCH, MCHC, or PLT point to the U-shaped linking only? These mechanisms will certainly be the subject of numerous scientific studies for a long time to come.

The limitations of this statistical analysis may also be acknowledged. One of them may be to consider all causes of death within one month of follow-up. The lack of detailed analysis of patient mortality was associated with limiting the research framework and not producing an excessive number of statistical results. On the other hand, at the beginning of the statistical analysis, we wanted to unify the research group and maintain “chronic” hematological measurements, and to exclude “emergency” cases, i.e., those associated with inflammation, e.g., after various types of injuries (hence, we decided to disable several classes of clinical diagnoses according to ICD10). We trust, however, that the research material screened repeatedly according to the adopted criteria could only increase the level of reliability of the obtained statistical results, thus allowing the following scientific conclusions to be drawn.

## 6. Conclusions

Based on the performed hematological, clinical, and statistical analyses of patients, we concluded that:

- there are likely relationships between blood counts and expected patient mortality at some time interval from measurements
- up to 1 month of observation since the first measurement of a hematological biomarker, RDW and MPV stand for a strong relationship with acute mortality of patients, whereas MCV, MCH, MCHC, and PLT show a U-shaped association
- RDW and MPV can be established as stronger predictors of early deaths of patients, MCV only in the highest levels (>95 fL), whereas MCH, MCHC, and PLT have no impact on the excess acute mortality in ED patients
- the statistical results obtained should be verified in further studies

## Figures and Tables

**Table 1:** Hematological biomarkers, categories of biomarkers, levels, and ranks of acute mortality (panel A); SMRs with 95% CI and *P* values (panel B).

		Panel A		Panel B		
Biomarker	Category	Level	% of death	SMR	95% CI	*P* value
RDW (%)	1	<12.5	2.9	1.00 (ref.)		
	2	12.5-12.9	3.1	0.91	(0.64, 1.28)	0.5730
	3	13.0-13.4	4.8	1.32	(0.97, 1.78)	0.0728
	4	13.5-13.9	7.9	1.96	(1.45, 2.63)	<0.0001
	5	14.0-14.4	7.6	1.88	(1.36, 2.59)	0.0001
	6	14.5-14.9	11.4	2.68	(1.98, 3.64)	<0.0001
	7	15.0-15.4	13.2	3.07	(2.20, 4.29)	<0.0001
	8	15.5-15.9	14.1	3.32	(2.36, 4.69)	<0.0001
	9	≥16.0	20.6	4.55	(3.49, 5.92)	<0.0001

MPV (fL)	1	<9.0	4.2	1.00 (ref.)		
	2	9.0-9.4	5.7	1.37	(0.83, 2.25)	0.2187
	3	9.5-9.9	6.3	1.49	(0.93, 2.37)	0.0980
	4	10.0-10.4	6.5	1.51	(0.95, 2.40)	0.0803
	5	10.5-10.9	8.1	1.82	(1.15, 2.88)	0.0110
	6	11.0-11.4	8.8	1.96	(1.23, 3.11)	0.0047
	7	11.5-11.9	10.0	2.15	(1.34, 3.45)	0.0015
	8	12.0-12.4	11.6	2.44	(1.50, 3.98)	0.0003
	9	12.5-12.9	12.3	2.56	(1.50, 4.36)	0.0005
	10	≥13.0	11.8	2.40	(1.38, 4.20)	0.0021

MCV (fL)	1	<75	12.4	1.00 (ref.)		
	2	75-79.9	6.7	0.59	(0.39, 0.90)	0.0138
	3	80-84.9	5.6	0.52	(0.37, 0.73)	0.0001
	4	85-89.9	6.5	0.57	(0.42, 0.78)	0.0005
	5	90-94.9	8.0	0.67	(0.48, 0.92)	0.0133
	6	95-99.9	15.0	1.18	(0.84, 1.66)	0.3402
	7	≥100	29.0	1.99	(1.40, 2.82)	0.0001

MCH (fmol)	1	<27.0	12.4	1.00 (ref.)		
	2	27.0-27.9	9.6	0.81	(0.61, 1.06)	0.1270
	3	28.0-28.9	8.1	0.70	(0.55, 0.89)	0.0029
	4	29.0-29.9	7.0	0.62	(0.50, 0.77)	<0.0001
	5	30.0-30.9	6.1	0.54	(0.44, 0.68)	<0.0001
	6	31.0-31.9	7.1	0.64	(0.51, 0.80)	0.0001
	7	32.0-32.9	7.8	0.69	(0.53, 0.90)	0.0062
	8	33.0-33.9	8.0	0.73	(0.52, 1.02)	0.0675
	9	≥34.0	14.4	1.22	(0.93, 1.60)	0.1572

MCHC (mmol/L)	1	<31.0	29.5	1.00 (ref.)		
	2	31.0-31.9	23.5	0.81	(0.62, 1.05)	0.1060
	3	32.0-32.9	12.1	0.46	(0.37, 0.59)	<0.0001
	4	33.0-33.9	9.0	0.37	(0.30, 0.46)	<0.0001
	5	34.0-34.9	5.6	0.25	(0.20, 0.32)	<0.0001
	6	35.0-35.9	4.1	0.20	(0.16, 0.26)	<0.0001
	7	36.0-36.9	4.3	0.23	(0.16, 0.33)	<0.0001
	8	≥37.0	4.1	0.21	(0.11, 0.39)	<0.0001

PLT (10^9^/L)	1	<100	28.4	1.00 (ref.)		
	2	100-149	12.3	0.46	(0.36, 0.59)	<0.0001
	3	150-199	7.8	0.30	(0.24, 0.38)	<0.0001
	4	200-249	5.9	0.24	(0.20, 0.31)	<0.0001
	5	250-299	5.2	0.23	(0.18, 0.29)	<0.0001
	6	300-349	7.5	0.32	(0.25, 0.42)	<0.0001
	7	350-399	8.8	0.37	(0.27, 0.51)	<0.0001
	8	400-449	9.6	0.39	(0.27, 0.57)	<0.0001
	9	≥450	13.3	0.51	(0.37, 0.69)	<0.0001

**Table 2:** The estimates of linear and quadratic regression coefficients for hematological biomarkers with 95% CI, *P* values *R*2 statistic.

Regression	Parameter	Mean	95% CI	*P* value	*R*2
Linear	RDW (%)	0.453	(0.416, 0.489)	<0.0001	0.9866
	Intercept	0.941	(0.779, 1.103)	<0.0001	0.9524
	MPV (fL)	0.169	(0.143, 0.195)	<0.0001	

Quadratic	Intercept	1.617	(1.371, 1.864)	0.0002	0.9843
	MCV (fL)	-0.712	(-0.853, -0.570)	0.0006	
	MCV (fL)^2^	0.108	(0.091, 0.125)	0.0003	
	Intercept	1.303	(1.105, 1.502)	<0.0001	0.8936
	MCH (fmol)	-0.308	(-0.399, -0.217)	0.0006	
	MCH (fmol)^2^	0.032	(0.023, 0.041)	0.0004	
	Intercept	1.334	(1.209, 1.459)	<0.0001	0.9838
	MCHC (mmol/L)	-0.346	(-0.410, -0.283)	0.0001	
	MCHC (mmol/L)^2^	0.026	(0.019, 0.033)	0.0007	
	Intercept	1.157	(0.868, 1.446)	0.0002	0.8170
	PLT (10^9^/L)	-0.342	(-0.474, -0.209)	0.0023	
	PLT (10^9^/L)^2^	0.031	(0.018, 0.044)	0.0034	

## Data Availability

The data used in the study are collected in a spreadsheet file and can be accessed together with R codes upon request.

## Abbreviations

CBC: Complete blood count
CIS: Cerebral ischemic stroke
CKD: Chronic kidney disease
COPD: Chronic obstructive pulmonary disease
CV: Composite cardiovascular
CVD: Cardiovascular disease
ED: Emergency department
EDTA: Ethylenediaminetetraacetic acid
GRACE: Global Registry of Acute Coronary Events
HR: Hazard ratio
ICD-10: International Statistical Classification of Diseases and Related Health Problems after 10^th^ Revision
IHD: Ischemic heart disease
MCH: Mean cell hemoglobin
MCHC: Mean cell hemoglobin concentration
MCV: Mean corpuscular volume
MPV: Mean platelet volume
NSTEMI: Non-ST-segment elevation myocardial infarction
PLT: Platelet
RDW: Red cell distribution width
SMR: Standardized mortality ratio
VAF: Vascular access failure.
